# Breast Cancer Cell-Derived Adenosine Enhances Generation and Suppressor Function of Human Adaptive Regulatory T Cells

**DOI:** 10.3390/jpm11080754

**Published:** 2021-07-30

**Authors:** Magis Mandapathil, Miroslaw J. Szczepanski, Edwin K. Jackson, Stephan Lang, Theresa L. Whiteside

**Affiliations:** 1 Department of Otorhinolaryngology, Asklepios Clinic St. Georg, 20099 Hamburg, Germany; 2 Department of Otorhinolaryngol Head & Neck Surg, Philipps University of Marburg, 35033 Marburg, Germany; 3 Department of Biochemistry, Medical University of Warsaw, PL-02097 Warsaw, Poland; mszczepanski@wum.edu.pl; 4 Department of Pharmacology, University of Pittsburgh, Pittsburgh, PA 15219, USA; ekj@pitt.edu; 5 Department of Otorhinolaryngology, University of Duisburg-Essen, 45147 Essen, Germany; s.lang@ukessen.de; 6 UPMC Hillman Cancer Center, Department of Pathology, University of Pittsburgh School of Medicine, Pittsburgh, PA 15213, USA; whitesidetl@upmc.edu

**Keywords:** Treg, adenosine, CD73, breast cancer (BrCa), immunosuppression

## Abstract

Introduction: Adaptive regulatory T cells (Tr1) are induced in the periphery by environmental stimuli. CD73 expression and adenosine (ADO) production by tumor cells may influence Tr1 generation and their immunosuppressive activity. Material and Methods: Tr1 were generated in co-cultures of CD4+CD25neg T cells, autologous immature dendritic cells (iDC), and irradiated ADO-producing CD73+ or non-producing CD73neg breast cancer (BrCa) cell lines (TU). The expression of ectonucleotidases and other surface markers on Tr1 was determined by flow cytometry. Tr1-mediated suppression of proliferation was evaluated in CFSE-based assays. Luciferase-based ATP detection assays and mass spectrometry were used to measure ATP hydrolysis and ADO levels. Cytokine levels were measured by ELISA or Luminex. CD73 expression on tumor cells or T cells in TU tissues was assessed by immunofluorescence. Results: CD73+ TU induced higher numbers of Tr1 cells (*p* < 0.01) than CD73neg TU. Tr1TU73+ hydrolyzed more exogenous ATP, produced more ADO, and mediated higher suppression than Tr1TU73neg (*p* < 0.05 for all). ARL67156, an ectonucleotidase inhibitor, and ZM241385, A2A receptor antagonist, reduced suppression of proliferation mediated by Tr1TU73+ cells (*p* < 0.01). Basal-like primary BrCa cells expressed higher levels of ectonucleotidases and induced more Tr1 than less aggressive primary luminal-like BrCa. Conclusion: BrCa producing ADO (CD73+ TU) favor the induction of Tr1, which expresses CD39 and CD73, hydrolyzes ATP to ADO, and effectively suppresses anti-tumor immunity.

## 1. Introduction

Human tumors develop a variety of mechanisms that down-regulate anti-tumor responses promoting tumor escape from the host immune system [1]. Similar to other solid tumors, breast cancers (BrCa) create a tumor microenvironment enriched in various immunosuppressive factors such as adenosine (ADO), which favor tumor growth by reducing anti-tumor immune responses [2]. ADO, a purine nucleoside, may be present at high concentrations in tumor tissues and body fluids [3]. ADO extracellular concentrations rise dramatically in response to tissue damage, hypoxia, or increased ATP levels, conditions commonly present in solid tumors [4]. Extracellular ADO is a product of ATP hydrolysis by ectonucleotidases, CD39, an ectonucleotidase triphosphate diphosphohydrolase (NTPDase), and CD73, a 5′ectonucleotidase. Ectonucleotidases are expressed by different tumors and presumably protect tumor cells from ATP-mediated toxicity [5]. CD73 expression by tumors has been linked to tumor progression, chemotaxis, migration, invasion, and metastasis formation [6]. 

ADO is one of the strongest immunosuppressive autacoids and its various signaling pathways in regulating innate and adaptive immune responses have been described in recent years [7]. It signals via cell membrane-associated receptors (A_1_, A_2A_, A_2B_ and A_3_), which are widely distributed in tissues. Immunosuppressive effects of ADO are largely mediated through A_2A_ receptors (A_2A_R) expressed on CD4^+^effector T cells (Teff). A_2A_R signaling leads to an intracellular upregulation of cAMP, which inhibits cell proliferation, cytokine production, including that of IL-2, IL-4, and IFNγ, and effector functions of CD8^+^ T cells [8,9]. ADO can also interfere with antigen-presenting functions of dendritic cells (DC), natural killer (NK) cell activity, B-cell functions, and myeloid cells [7,10]. Further, ADO might directly stimulate tumor growth or promote tumor growth by suppression of anti-tumor immunity [11,12]. 

We and others have reported that human naturally occurring regulatory T cells (nTreg) as well as adaptive Treg (Tr1), which accumulate in the tumor microenvironment, express ectonucleotidases and hydrolyze extracellular ATP, thus contributing to elevated levels of ADO [13,14,15,16,17]. It is, therefore, possible to speculate that tumor- or Treg-derived ADO promotes the generation of adaptive Treg (Tr1) in the tumor microenvironment. 

Here, we test the hypothesis that BrCa-derived ADO promotes Tr1 induction. Tr1 are induced in the periphery in response to local stimuli and mediate cell-independent suppression of Teff functions via soluble immunosuppressive factors [18,19]. Tr1 may be generated from nTreg or conventional CD4 T cell (Tconv) conversion, but neither their origin nor mechanisms involved in their generation are known. Using a Tr1 in vitro generation model [20] developed in our laboratory and modified to include an ADO-producing CD73^+^ BrCa cell line or a CD73^neg^ BrCa cell line unable to produce ADO, we compare the phenotype and functions of Tr1 generated under different conditions. BrCa-derived ADO is shown to play a key role in Tr1 induction and their suppressive functions. 

## 2. Materials and Methods

### 2.1. Tumor Cell Lines

Human BrCa cell lines MDA-MB231, MDA-MB435, BT-20, and SUM149 are basal-like, and MCF-7 and T470 are luminal-like. All were established from primary tumors and obtained from the ATCC. Tumor cells were cultured in DMEM supplemented with 10% (*v*/*v*) FCS, 100IU/mL penicillin, 100 μg/mL streptomycin, and 2 mmol/L L-glutamine (all from Invitrogen) at 37 °C in the atmosphere of 5% CO_2_ in air.

### 2.2. Collection of Peripheral Blood Mononuclear Cells (PBMC)

Blood samples (30–50 mL) from 10 healthy donors were drawn into heparinized tubes and centrifuged on Ficoll-Hypaque gradients (GE Healthcare Bioscience, Bath, UK). PBMC were recovered, washed in AIM-V medium (Invitrogen, Waltham, MA, USA), counted in a trypan blue dye, and immediately used for experiments.

### 2.3. Co-Cultures for Tr1 Generation

Tr1 was generated using a modified co-culture system [18]. Briefly, monocytes were isolated via adherence to plastic and were differentiated into iDC by culture in AIM-V medium supplemented with GM-CSF (1000 IU/mL) and IL-4 (4 ng/mL) for 7 days. CD4^+^CD25^neg^ cells were isolated from the lymphocyte fraction using the Regulatory T cell Isolation Kit (Miltenyi Biotech, Bergisch Gladbach, Germany) and AutoMACS. Cell aliquots (1 × 10^6^) were co-incubated in flat-bottom 24-well plates with autologous iDC (1 × 10^5^) and irradiated (3000 rad) tumor cells (1 × 10^5^) using complete AIM-V supplemented with IL-2 (10 IU/mL), IL-10 (20 IU/mL) and IL-15 (20 IU/mL). The medium was changed on days 3 and 6. On day 9, it was replaced by fresh medium containing OKT-3 (1 μg/mL) and brefeldin A (1 μg/mL). On day 10, lymphocytes and cell supernatants were separately harvested. To some co-cultures, α,β-methylene ADP (100 μg/mL) was added on days 0, 3, and 6. In other experiments, CD4^+^CD25^neg^ cells were incubated with 2-chloroadenosine (CADO, 6 μM) in the presence of IL-2, IL-10, and IL-15 but in the absence of tumor or iDC. In control cultures, CD4^+^CD25^neg^ T cells were cultured for 10 days in a complete medium supplemented with 150 IU/mL IL-2 in 24-well plates at 37 °C in the atmosphere of 5% CO_2_ in air.

### 2.4. Antibodies

The following anti-human monoclonal antibodies (mAbs) were used for flow cytometry: anti-CD3-ECD, -CD4-ECD, -CD4-PC5, -CD25-PE, -GITR-FITC, -FOXP3-FITC, -CD39-FITC, -CD39-PE, -CD73-PE, -CD122-FITC, -CD132-FITC, -TGFβ-PE, -IL-10-PE, and -CTLA4-PE. All mAbs and their respective isotypes, which served as negative controls for surface and intracellular staining, were purchased from Beckman Coulter, except for antibodies to FOXP3, and CD39, which were purchased from eBioscience. Anti-CD73-PE and anti-IL-10 were purchased from BD Pharmingen and antibodies to GITR, TGF-β, CD132, and CTLA^4^ from R&D Systems. Before use, all antibodies were titrated using resting as well as activated PBMC.

### 2.5. Surface and Intracellular Staining

Tumor cells, isolated T cells from co-cultures or control cells, were stained for flow cytometry as previously described [20]. Briefly, cells were incubated with the mAbs for surface markers for 30 min at 4 °C in the dark and then fixed with 2% (*w*/*v*) paraformaldehyde in PBS for 15 min. Afterward, cells were permeabilized with 0.1% (*w*/*v*) saponin and stained with Abs specific for intracellular markers for 30 min at 4 °C in the dark. Cells were washed twice with 0.1% saponin in PBS, resuspended in a flow solution, and analyzed by flow cytometry. Appropriate isotype controls were always included.

### 2.6. Flow Cytometry

Flow cytometry was performed using an EPICS^®^ XL-MCL flow cytometer equipped with Expo32 software (Beckman Coulter, Brea, CA, USA). The acquisition and analysis gates were restricted to the lymphocyte gate based on cell properties in the forward (FSC), and side scatter (SSC). FSC and SSC were set on a linear scale, and at least 10^6^ cells were acquired. Analysis was performed using the Coulter EXPO 32vl.2 program (Brea, CA, USA). For additional analyses, gates were restricted to the CD3^+^CD4^+^ cell subset. For analysis of tumor cells, SSC was set on a logarithmic scale.

### 2.7. Suppression Assays

MACS-isolated autologous CD4^+^CD25^neg^ responder cells (RC) were incubated with 1.5 μM CFSE (Molecular Probes) for 15 min at 37 °C. Unbound CFSE was quenched by adding an equal volume of FCS. Cells were washed with PBS, aliquoted into wells of flat-bottom 96-well plates (10^5^ cells/well), and co-incubated with autologous suppressor cells (SC) obtained from co-cultures or T cells from control cultures. The ratios of RC:SC were 1:1 and 1:2. In some experiments, inhibitors or modifiers were added to RC prior to the SC addition. These were: ARL67165 (250 μM) and adenosine 5’-(α,β-methylene) diphosphate (100 μM), both purchased from Sigma-Aldrich; ZM241385 (0.3 μM) purchased from Tocris Bioscience; IL-10 or TGF-β neutralizing Abs purchased from R&D Systems (Minneapolis, MN, USA). RC were stimulated with plate-bound OKT-3 (2 μg/mL) and soluble anti-CD28 mAb (2 μg/mL; Miltenyi Biotech) in the presence of 150 IU/mL IL-2 for 5 days. All CFSE data were analyzed using the ModFit software provided by Verify Software House (Topsham, ME, USA). The percentage of suppression was calculated as previously described [16].

### 2.8. siRNA Knockdown of Endogenous CD73

CD73 expression in BrCa cell lines was transiently silenced using small interfering RNAs (siRNA). Transfection with siRNA was performed according to the manufactures’ instructions (Santa Cruz Biotechnology, Inc., Dallas, TX, USA). Optimal siRNA concentrations were determined in preliminary titrations using flow cytometry to measure down-regulation of CD73 expression in tumor cells. Briefly, 2 × 10^5^ tumor cells were seeded in wells of six-well plates and adhered for 24 h in antibiotics-free medium. Cells were resuspended in transfection medium containing CD73 siRNA (100 pM) or control non-targeting siRNA and incubated for 24 to 96 h at 37 °C in the atmosphere of 5% CO2 in air. Expression of CD73 was tested using flow cytometry, immunofluorescence, or Western blots. ADO production was measured by mass spectrometry. 

### 2.9. CD73 Transfection

CD73 was transfected into CD73neg BrCa cell lines using a human cDNA ORF clone purchased from Origene, Rockville. Transfection was performed according to the manufactures’ instructions (Invitrogen, Waltham, MA, USA). Optimal plasmid concentrations were determined in preliminary titrations using flow cytometry to measure upregulation of CD73 expression in tumor cells. Briefly, 2 × 10^5^ tumor cells were seeded in wells of six-well plates and adhered for 24 h in antibiotics-free medium. Cells were resuspended in transfection medium containing CD73 plasmid or control non-targeting siRNA and incubated for 24 to 72 h at 37 °C in the atmosphere of 5% CO_2_ in air. Expression of CD73 was tested using flow cytometry, immunofluorescence, or Western blots. ADO production was measured by mass spectrometry.

### 2.10. Western Blots

Whole-cell protein extracts were prepared from BrCa cell lines. Cells were washed twice and lysed at 4 °C in a lysis buffer containing 0.5% NP40, 150 mM NaCl, and 50 mM Tris base (Sigma, Darmstadt, Germany). Laemmli loading buffer (4% SDS, 10% b-mercaptoethanol 20% glycerol, 0.004% bromophenol blue, 0.125 M Tris HCl) was added to the cell lysates at a 1:1 ratio (*v*/*v*), and the lysates were then boiled for 5 min. Protein extracts were subjected to electrophoresis on 4%–15% Tris-HCl gradient gels (BioRad, Hercules, CA, USA) and were subsequently transferred to polyvinylidene fluoride membranes (PVDF Millipore). The membranes were treated with rabbit polyclonal primary antibodies specific for human CD73 (Santa Cruz, 1:250) followed by goat anti-rabbit secondary antibody conjugated to horseradish peroxidase (Pierce, goat, 1:150,000). SuperSignal West Femto Maximum Sensitivity Substrate (Pierce) and Kodak BioMax MR Film were used to visualize the target protein.

### 2.11. ATP Hydrolysis Assay

Tumor cells or T cells obtained from the co-cultures or control cultures were incubated in wells of flat-bottom 96-well plates (25 × 10^3^/well) for 30 min with 10 μM ATP (Sigma-Aldrich, Darmstadt, Germany). Some wells were pre-incubated with ARL67156, at the final concentration of 250 μM for 30 min prior to ATP addition. The concentration of “unhydrolyzed” ATP was determined by measuring the frequency of luminescent events (counts per minute) in the luciferase-based detection system (ATP Lite Luminescence ATP Detection Assay System; PerkinElmer, Waltham, MA, USA). The average cpm was determined from triplicate wells.

### 2.12. Mass Spectometric Analysis of Adenosine Production

Tumor cells or T cells obtained from co-cultures or control cultures were incubated with 10 μM exogenous ATP or 100 μM AMP in wells of 96-well flat-bottom plates (25,000/well) for various time periods. Cell supernatants were collected, boiled for 2 min, and stored on dry ice until analysis. ADO levels were measured by high-performance liquid chromatography-tandem mass spectrometry using a triple quadrupole mass spectrometer (TSQ-Quantum-Ultra, Thermo Fisher Scientific, San Jose, CA, USA) operating in the selective reaction monitoring mode with a heated electrospray ionization source as previously described [18]. The average concentration of ADO was determined in duplicate wells. 

### 2.13. ELISA and Luminex

Supernatants of co-cultures or of RC cultured alone (controls) were harvested on day 5 for cytokine assays. T cells in co-cultures were supplemented with fresh medium containing OKT-3 (1 μg/mL) and anti-CD28 (1 μg/mL) on day 9 and 24 h after supernatants were collected and stored frozen until analysis. Levels of TGF-β in acidified supernatants were analyzed by ELISA (R&D Systems). Levels of IL-2, IFN-γ, and IL-10 were tested by Luminex using a human cytokine 10-plex Ab bead kit (Biosource/Invitrogen). All assays were performed according to manufacturers’ instructions.

### 2.14. Immunofluorescence

Luminal (n = 18) and basal (n = 14) BrCa tumor tissue samples obtained from the Magee Health Sciences Tissue Bank and the University of Medical Science Poznan, Poland, were embedded in OCT, and 5 mm frozen sections were cut in a cryostat, fixed for 10 min in cold acetone/ethanol (1:1) and dried at room temperature (RT). The following anti-human Abs were used for staining: anti-CD4-FITC, anti-CD25-PE (BD Pharmingen, San Diego, CA, USA), anti-CD39, anti-CD73 (Santa Cruz), and anti-FOXP3-FITC (eBioscience, San Diego, CA, USA). The secondary Ab was Cy5-labeled donkey anti-rabbit (Jackson Immuno Research, West Grove, PA, USA). To eliminate non-specific staining, tissue sections were incubated with 10% donkey serum for 1 h and then washed in PBS. Sections were incubated with primary Abs for 1 h in a moist chamber at RT. Next, slides were washed and incubated with the secondary Abs under the same conditions. Primary Abs were omitted in all negative controls. Sections were mounted in a mounting medium with DAPI (Vector Laboratories, Burlingame, CA, USA) in order to trace cell nuclei. Slides were evaluated in the Olympus Provis (Olympus, Tokyo, Japan) fluorescence microscope under 400× *g*. For digital image analysis, the software Adobe Photoshop 6.0 version was used. The number of CD4+ T cells positive for Foxp3, CD39, or CD73 were analyzed in seven randomly selected view points of each tumor specimen, and means and SD were calculated. 

### 2.15. Statistical Analysis

All data were presented as the means of at least three experiments ± 1 SD. The data were analyzed using the Student’s *t*-test, and *p*-values ≤ 0.05 were considered to be significant.

## 3. Results

### 3.1. CD73 Expression and Adenosine Production by BrCa Cell Lines

**Figure 1:**
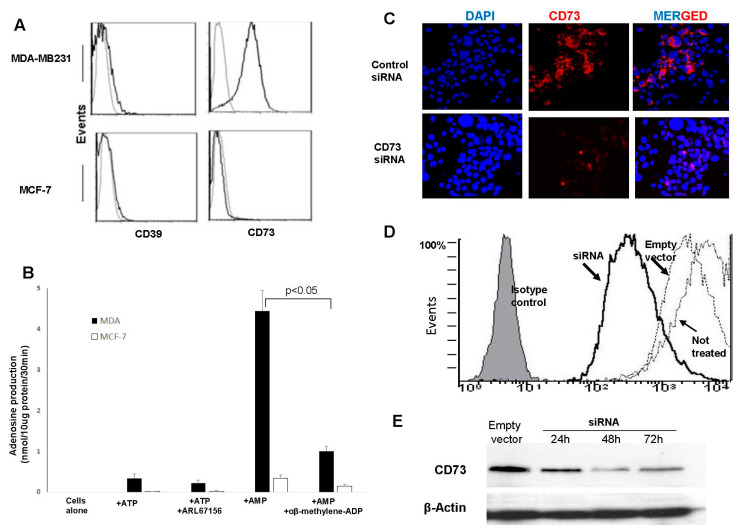
Ectonucleotidase expression and function in MDA-MB231 versus MCF-7 cell lines. (**A**) Tumor cells were stained for CD39 and CD73 and analyzed by flow cytometry. One representative experiment of 3 performed is shown. (**B**) MDA-MB231 and MCF-7 tumor cell lines were incubated with exogenous ATP or AMP in the presence or absence of ARL67156, a CD39 inhibitor, or αβ-methylene-ADP, a CD73 inhibitor. Levels of generated adenosine were measured by mass spectrometry. Data are from 3 independent experiments and show means ± SD. (**C**) MDA-MB231 cells were treated with siRNA specific for CD73 or with scrambled siRNA as control for 24 h. Cells were layered on slides, and after fixation with methanol were stained with Abs specific for CD73. Tumor cells treated with the scrambled siRNA were positive for CD73, while those treated with CD73-specific siRNA were negative. (Mag × 400). (**D**) Flow cytometry analysis of MDA-MB231 cells treated with CD73-specific siRNA or scrambled siRNA for 24 h. Loss of CD73 expression in cells treated with CD73-specific siRNA is evident. Isotype control IgG is shown as a gray histogram. (**E**) Western blot showing a partial decrease in CD73 after 24, 48, and 72 h of treatment of MD-MB231 cells with CD73-specific siRNA. The densitometry readings in pixels were: empty vector = 127,942; siRNA 24 h = 57,708; siRNA 48 h = 12,748; and siRNA 72 h = 52,950. Results are representative of one experiment of 5 performed.

The expression of CD73 and CD39 on the surface of seven BrCa cell lines was evaluated by flow cytometry. Only those lines derived from basal ductal BrCa, (MDA-MB231, MDA-MB231, BT-20, and SUM 149) were variably positive for CD73 (58%–98% positive cells). Cell lines derived from a luminal ductal BrCa (MCF-7, SKBR3, and T47D) were negative for CD73 (see Figure 1A for MCF-7). CD39 was not detected on BrCa cell lines (e.g., Figure 1A). MDA-MB231 (98% CD73^+^) and MCF-7 (CD73^neg^) cell lines were selected for further studies as the representative BrCa cells for surface expression of CD73 or its absence, respectively. 

Tumor cells were analyzed for CD73 enzymatic activity after incubation with exogenous AMP. MDA-MB231 but not MCF-7 cells produced ADO, and α,β-methylene-ADP, a CD73 inhibitor, blocked ADO production. Incubation of MDA-MB231 cells with exogenous ATP did not result in ADO production (Figure 1B). We used siRNA to knock down CD73 in MDA-MB231 cells (Figure 1C–E). The endogenous CD73 protein level was decreased after 48 h treatment with CD73-specific siRNA (Figure 1C,D). In Western blots, the CD73 protein level was transiently decreased after 48 h treatment with CD73-specific siRNA relative to that in control cells (Figure 1E). ADO production was also significantly reduced in tumor cells exposed to CD73-specific siRNA for 24 h, as determined by mass spectrometry (from 2753 ± 1143 to 182 ± 65 pg/μL; means ± SD from three experiments; *p* < 0.01). These experiments confirmed that ADO production by tumor cells was dependent on CD73 expression and its enzymatic activity.

### 3.2. Phenotypic Analysis of In Vitro-Generated Tr1 Cells

**Figure 2:**
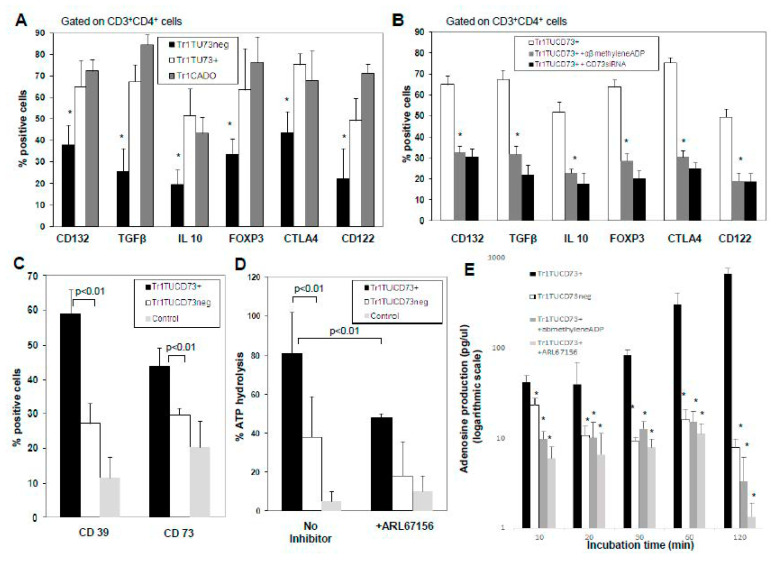
Phenotypic characterization and ectonucleotidase expression and activity in generated Tr1. (**A**) Flow cytometry analysis of Tr1 generated in the presence of a CD73^+^ (TrlTU73^+^) or CD73^neg^ (Tr1TU73^neg^) tumor cell lines or exogenous CADO (6 μM). CD4^+^CD25^neg^ T cells cultured for 10 d in the presence of IL-2 (150 IU/mL) but in the absence of tumor cells and DC served as reference cells for all co-cultures. (**B**) Flow cytometry analysis of Tr1TU73^+^ generated in the presence of MDA-MB231 cell line with and without the addition of αβ-methylene-ADP, a CD73 inhibitor, or in the presence of the MDA-MB231 cell line treated with CD73 siRNA. Data are from 10 independent experiments and show means ± SD. (**C**) CD39 and CD73 expression of Tr1TU73^+^ or Tr1TU73^neg^ was measured by flow cytometry. CD4^+^CD25^neg^ T cells cultured for 10 d in the presence of IL-2 (150 IU/mL) served as control. (**D**) Tr1TU73^+^ or Tr1TU73^neg^ were plated in 96-well plates (25,000 cells/well) in serum-free medium with 10 μM of exogenous ATP. Unhydrolyzed ATP was measured after 30 min of incubation. The % hydrolysis was calculated based on a standard curve. ARL67156 (an ecto-ATPase inhibitor) was added to selected wells. Data (means ± SD) are from 10 independent experiments. (**E**) Adenosine production by Tr1TU73^+^ or Tr1TU73^neg^ was determined by mass spectrometry. In some experiments, α,β methylene ADP, a CD73 inhibitor, α,β methylene ADP or ARL67156, a CD39 inhibitor, was added. Data show one representative experiment of 3 performed. In (**A**–**E**), asterisks * indicate differences at *p* < 0.01 to *p* < 0.05.

Using the co-culture system, Tr1 was generated from autologous CD4^+^CD25^neg^ T cells by co-incubation with irradiated MDA-MB231 (CD73^+^) or MCF-7 (CD73^neg^) tumor cells and autologous iDC. The phenotype of T cells proliferating in these co-cultures was consistent with that previously determined for Tr1: CD25^low^, FOXP3^+/low^, CD122^+^, CD132^+^, TGFβ^+^, and IL-10^+^ [20]. Tr1 generated in co-cultures established with ADO-producing CD73^+^ BrCa cells (Tr1TU73^+^) contained a significantly higher proportion of cells expressing these markers than Tr1 in co-cultures with CD73^neg^ tumor cells (Tr1TU73^neg^) (Figure 2A). Similar results were obtained by using other CD73^+^ or CD73^neg^ BrCa cell lines (data not shown). 

To confirm the hypothesis that ADO present in these co-cultures plays a role in Tr1 induction, the co-cultures were established in the absence of tumor cells but in the presence of exogenous CADO, a synthetic ADO analog. Adding CADO on days 0, 3, 6, and 9 to co-cultures promoted Tr1 generation, which was comparable to that in co-cultures containing CD73^+^ tumor cells (Figure 2A). Control cultures without CADO contained few Tr1 (data not shown). When α,βmethylene-ADP was added to co-cultures with CD73^+^ MDA-MB231 BrCa cells, the frequency of Tr1 was significantly reduced but not entirely blocked (Figure 2B). In addition, supernatants of Tr1 generated in the presence of CD73^+^ MDA-MB231 cells or CADO contained the highest ADO levels (78 ± 50 pg/mL and 178 ± 101 pg/mL vs. 13 ± 5 pg/mL in supernatants of MCF-7 (CD73^neg^) cells). To further confirm that CD73 expression and ADO production by BrCa cells directly contribute to Tr1 expansion, siRNA was used to knock down CD73 in MDA-MB231 (Figure 2B). The frequency of Tr1 was significantly reduced in co-cultures established in the presence of CD73-silenced MDA-MB231or when α,βmethylene-ADP was used (Figure 2B). Next, we transfected CD73 into the CD73neg tumor cell line (MCF-7) and used these cells in the co-culture system to generate Tr1. As shown in Figure 1, transfection of CD73 resulted in an upregulation of Tr1 generation and expression of relevant Tr1 markers. 

### 3.3. Ectonucleotidase Expression and Activity of Tr1

The majority of Tr1 generated in co-cultures with MDA-MB231 tumor cells expressed CD39 and CD73 (Figure 2C). However, the percentages of CD39^+^ and CD73^+^ Tr1TU73^+^ were significantly higher than those of Tr1 generated in the presence of CD73neg BrCa or control cells (Figure 2C). We also confirmed the ability of Tr1 cells generated in co-cultures to hydrolyze ATP. As shown in Figure 2D, Tr1TU73^+^ hydrolyzed more exogenous ATP than Tr1TU73^neg^ or control cells (*p* < 0.01). In the presence of ARL67156, an inhibitor of CD39, ATP hydrolysis was reduced (*p* < 0.01) (Figure 2D). 

### 3.4. ADO Production by Tr1

The ability of Tr1TU73^+^ vs. Tr1TU73^neg^ to produce ADO following the addition of exogenous ATP was evaluated. Tr1TU73^+^ produced more ADO than Tr1TU73^neg^ (Figure 2E). ADO production was almost completely blocked byARL67156 (data not shown). In addition, α,β-methylene-ADP, a selective CD73 inhibitor, completely inhibited ADO production by these (Figure 2E).

### 3.5. Cytokine Production by Tr1

Cytokine levels in supernatants of TU CD73^+^ vs. TU CD73^neg^ stimulated with OKT-3 and anti-CD28 mAb cells were analyzed by Luminex and ELISA. Tr1TU73^+^ produced significantly higher levels of IL-10 and TGFβ_1_ than Tr1TU73^neg^. In contrast, levels of IFN-γ in Tr1TU73^+^ supernatants were decreased compared to Tr1TU73^neg^ (Table 1A). α,β-methylene-ADP significantly blocked IL-10 and TGF-β1 production by Tr1, while enhancing that of IFN-γ (Table 1A).

### 3.6. Suppression Mediated by Tr1

**Figure 3:**
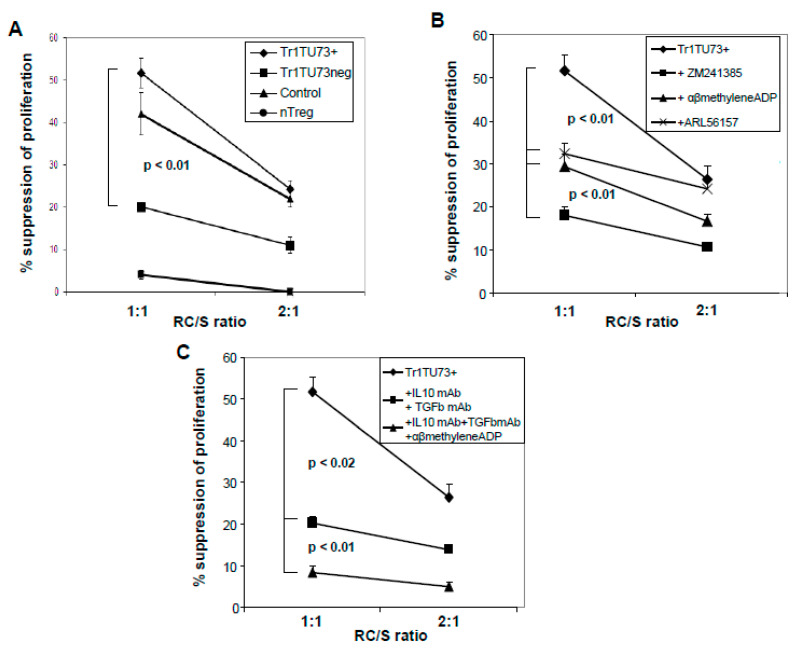
Suppression of responder cell (RC) proliferation mediated by Tr1TU73^+^ and Tr1TU73^neg^. Cells were analyzed by flow cytometry gating on CD4^+^CFSE^+^ T-cell subsets. (**A**) Percent suppression of CD4^+^CD25^neg^ cell proliferation mediated by Tr1TU73^+^ vs. Tr1TU73^neg^ at two different RC/S ratios is shown. CD4^+^CD25^neg^ T cells cultured for 10 d in the presence of IL-2 (150IU/mL) and nTreg served as control. (**B**,**C**) Tr1TU73^+^ were titrated into MACS-sorted CFSE-labeled CD4^+^CD25^neg^ cells (RC) in the presence or absence of various inhibitors as described in Materials and Methods. (**A**–**C**) data are means ± SD from 10 individual experiments.

Suppressor activity of Tr1TU73^+^ harvested from the co-cultures and incubated with autologous CFSE-labeled CD4^+^CD25^neg^ RC at the 1SC:1RC ratio was higher than that of Tr1TU73^neg^ (52% ± 3% vs. 20% ± 1%, *p* < 0.01; Figure 3A). Cells isolated from the same individual mediated lower suppression than Tr1, and cells from control cultures did not suppress proliferation of RC. These results indicate that Tr1TU73^+,^ which produces ADO, mediated higher suppression than Tr1TU73^neg^, which does not produce ADO. 

### 3.7. ADO Is Responsible for Tr1-Mediated Suppression of RC Proliferation

Since Tr1TU73^+^ produced ADO in the presence of exogenous ATP and also mediated suppression of RC proliferation, we asked whether ADO is directly responsible for this suppression. When ARL67156, a structural analog of ATP and CD39 inhibitor, was added to co-cultures of Tr1TU73^+^ with autologous CSFE-labeled RC at the concentration previously shown by us to block ectonucleotidase activity [13], suppression was inhibited (*p* < 0.01) compared to cells cultured in the absence of this inhibitor (Figure 3B). Similarly, the addition of α,β-methylene-ADP, an inhibitor of CD73, to co-cultures significantly reduced Tr1-mediated suppression of RC proliferation by Tr1TU733^+^ cells (Figure 3B). Because immunosuppressive effects of ADO are largely mediated via the A_2A_R expressed on Teff cells [15], blocking of A_2A_R on RC in the co-cultures was expected to down-regulate Tr1-mediated immunosuppression. The addition of ZM241385, a selective A_2A_ and A_2B_ receptor antagonist, two co-cultures reduced immunosuppression mediated by Tr1TU73^+^ at the 1S:1RC ratio (*p* < 0.01; Figure 3B). Since only partial inhibition of Tr1-mediated suppression was observed in the presence of these inhibitors, we considered the possibility that TGF-β and/or IL-10, known to be produced by Treg [18], were also in part responsible for suppression. The addition of neutralizing TGF-β or IL-10 mAb to Tr1-RC co-cultures resulted in a reduction (*p* < 0.02) of Tr1-mediated suppression (Figure 3C). In the presence of both neutralizing Abs plus α,β-methylene-ADP, an almost complete block of Tr1TU73^+^-mediated immunosuppression was observed at *p* < 0.01 (Figure 3C). In aggregate, these data suggest that while ADO is mainly responsible for Tr1-mediated immunosuppression, IL-10 and TGF-β may also contribute.

### 3.8. Levels of Cytokines and ADO in Tr1 Supernatants

To determine how the presence of Tr1 altered the cytokine profile of RC, supernatants of different Tr1 co-cultures with RC were tested for cytokine levels and compared to supernatants of RC cultured alone. Cultures of RC alone were negative for IL-10 and TGF-β but, as expected, were positive for IL-2 and IFN-γ. These supernatants contained very low levels of ADO (Table 1B). Significantly higher levels of IL-10, TGF-β_1,_ and ADO were detected in supernatants of RC co-cultured with Tr1TU73^+^, whereas levels of IL-2 and IFN-γ were low (Table 1B). When α,β-methylene-ADP was added to co-cultures, IL-10, TGF-β_1,_ and ADO levels were significantly reduced (*p* < 0.05), while IL-2 and IFN-γ levels increased (*p* < 0.05) (Table 1B). These results suggest that Tr1 generated in an ADO-enriched tumor microenvironment produces immunoinhibitory cytokines able to suppress RC proliferation. Inhibition of ADO production tended to increase the production of Th1-type cytokines.

### 3.9. In Situ Analysis of CD39 and CD73 in Basal and Luminal Ductal BrCa

**Figure 4:**
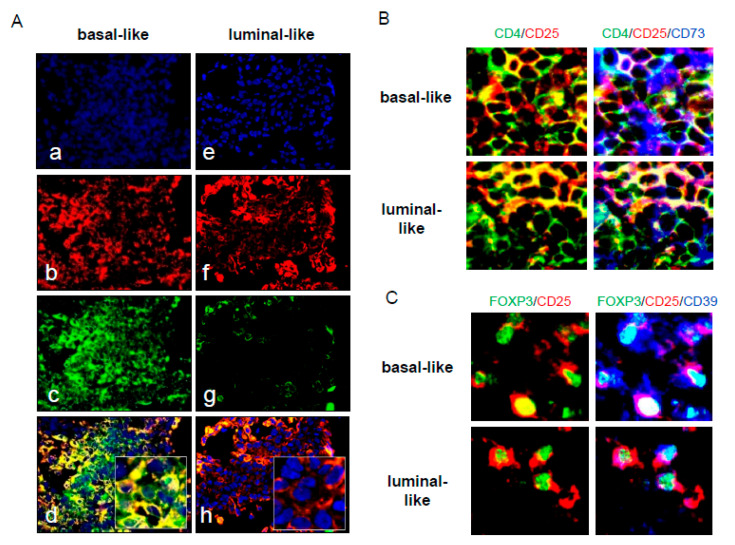
Phenotypic analysis of ectonucleotidase expressing tumor cells and Treg in tumor tissues of breast cancer patients. Breast cancer tissue biopsies from basal and luminal BrCa were examined using multicolor immunofluorescence and confocal microscopy. (**A**) Expression of CD73 in tumor cells of basal and luminal BrCa is shown in representative samples of 14 basal and 18 luminal individual tumor specimens examined (final Mag × 300). (**a**,**e**): DAPI staining; (**b**,**f**): cytokeratin staining; (**c**,**g**): CD73 staining; (**d**,**h**): merged images: tumor cells are red, CD73^+^ cells are green and CD73^+^ tumor cells appear yellow. Inserts in (**d**,**h**) show cells at Mag × 400. (**B**,**C**) CD4^+^CD25^+^CD73^+^ as well as FOXP3^+^CD25^+^CD39^+^ T cells are shown in sections of a representative tumor of 5 examined (Mag × 400). Sections are stained for CD4^+^, CD25^+^ or CD4^+^/CD25^+^/CD73^+^ cells and FOXP3^+^/CD25^+^ or FOXP3^+^/CD25^+^/CD39^+^ cells. In (**B**), CD4^+^ cells are green, CD25^+^ cells are red, CD4^+^CD25^+^ cells are yellow, while CD4^+^CD25^+^CD73^+^ cells appear pseudo-blue. In (**C**), FOXP3^+^ cells are green, CD25^+^ cells are red, FOXP3^+^CD25^+^ cells are yellow, and FOXP3^+^CD25^+^CD39^+^ cells appear pseudo-blue. Expression of ectonucleotidases on tumor-infiltrating Treg is higher in basal than luminal BrCa.

Since we have obtained evidence for a differential CD73 expression in basal-like and luminal-like BrCa cell lines, we suspected that CD73 expression in primary BrCa cells might also be different. When BrCa tissue biopsies (n = 32) were examined by multicolor immunofluorescence and confocal microscopy, we found that basal BrCa cells were strongly CD73^+^, whereas CD73 was not expressed on luminal BrCa (compare panels d and h in Figure 4A). Interestingly, this strong CD73 expression was seen in all basal BrCa tissues examined but not in tissues obtained from luminal BrCa. In addition, we examined the expression of CD39 and CD73 in Treg infiltrating the tumor tissues. As shown in Figure 4B, higher numbers of infiltrating Treg are seen in the microenvironment of basal tumors compared to that of luminal tumors (7.91 ± 1.17 vs. 3.9 ± 0.67 for CD4+ T cells positive for Foxp3). In addition, ectonucleotidase expression appeared to be increased in T cells infiltrating basal BrCa relative to luminal BrCa (7.65 ± 1.43 vs. 3.83 ± 0.42 for CD4+ T cells positive for CD39 and 15.9 ± 1.49 vs. 6.42 ± 0.63 for CD4+ T cells positive for CD73). These data suggest that in primary basal ductal BrCa cells, ADO-mediated immunosuppression may be enhanced because of enhanced ectonucleotidase expression relative to luminal ductal BrCa.

## 4. Discussion

Inflammatory cells infiltrating human tumor tissues include numerous Treg [20,21]. These Treg mediate potent immunosuppression [20] and phenotypically resemble Tr1 generated in the co-culture system [15]. Tumor cells that are known to produce a variety of immunosuppressive factors such as PGE_2_, ADO, IL-10, and TGF-β, appear to play a major role in the conversion of CD4^+^CD25^neg^ precursors to CD4^+^CD25^+^CD39^+^FOXP3^+^ Treg [22,23]. Not surprisingly, more invasive and less well-differentiated tumors have the potential to create a strongly suppressive microenvironment, effectively promoting Tr1 generation and accumulation. However, mechanisms used by the tumor to induce Tr1 and support their suppressive functions in vivo are not fully understood. ADO is recognized as an important regulator of anti-tumor immunity [4,5,7], and the expression of ectonucleotidases on tumor cells has been linked to poor outcomes [24]. For example, increased CD39 expression accompanied by enhanced ATP and ADP hydrolysis was reported to predict disease progression in chronic lymphocytic leukemia [25]. Furthermore, CD73 expression in tumor cells was reported to correlate with angiogenesis, metastasis, and tumor immune escape [6,7]. 

In this study, using human BrCa cell lines, which either express CD73 or not, we show that tumor-derived ADO promotes the outgrowth of immunosuppressive Tr1 in culture. These Tr1 themselves express ectonucleotidases, produce ADO, and use it to mediate suppression, as recently described by us [22]. In co-cultures containing exogenous ADO in place of CD73^+^ tumor cells, the outgrowth of immunosuppressive Tr1 was promoted, as was their activity. ADO significantly up-regulated surface expression of FOXP3, CTLA-4, IL-10, and TGF-β, molecules associated with immune suppression, in Tr1. In addition, as in mice, ADO binding to A_2_R on human effector T cells can cause energy and induce expression of FOXP3 [21]. Further, ADO-induced Tr1 expressed ectonucleotidases and used ADO to inhibit the proliferation and cytokine production of autologous Teff cells. In the presence of ectonucleotidase inhibitors, Tr1 outgrowth and their suppressor activity were significantly reduced but not eliminated, an indication that other suppressive molecules present in co-cultures also contribute to Tr1-mediated suppression. Tr1 is known to secrete IL-10 and TFG-β, and our experiments show that ADO, IL-10, and TGF-β are involved in Tr1-mediated suppression. In addition, other soluble factors such as Granzyme B (GrB) and perforin have been described as mediators of Tr1-driven immunosuppression [26,27].

To relate our in vitro model for Tr1 generation to events occurring in BrCa tissues, we evaluated the CD73 presence in situ. Despite a rather small sample size (n = 32), we found that predominantly basal and not luminal ductal BrCa cells predominantly expressed CD73 in tissue biopsies and thus were presumably able to produce immunosuppressive ADO. These preliminary results need to be further evaluated in a larger patient cohort to analyze the predictive role of ectonucleotidases/adenosine in the prognosis as well as therapeutic efficacy in breast cancer patients. It is well known that luminal ductal BrCa is less aggressive and has a significantly better prognosis than basal BrCa [28,29]. CD73 expression has been previously linked to tumor development, progression, aggressiveness, a pro-metastatic phenotype, and a loss of estrogen receptor-α in BrCa [30,31,32,33]. Thus, the ability to produce ADO and thus to promote Tr1 generation and Tr1-mediated immunosuppression appears to be an attribute of the more aggressive types of BrCa.

Based on our in vitro data, it seems reasonable to suggest that inhibition of ectonucleotidases and of the adenosinergic pathway operating in BrCa could antagonize tumor progression. Indeed, it has been shown in mice that evasion of tumor cells from the immune system and cancer progression is linked to the adenosinergic pathway [34]. A_2A_R-deficient mice showed enhanced anti-tumor immune responses and a significant reduction in tumor growth compared to WT animals [35]. Similarly, in CD39-deficient mice, and inhibition of tumor angiogenesis, endothelial and inflammatory cell migration into the tumor, and formation of pulmonary metastases, as well as the increase in NK cell numbers and Teff functions, was frequently observed [36,37]. In addition, anti-CD73 mAb treatment has been shown to reduce tumor growth and number of lung metastases in mice [24]. 

In summary, our data implicating ADO in Tr1 outgrowth and activity emphasize its key role in the immune regulation of BrCa. More aggressive breast tumors, which expressed CD73^+^ and produced ADO, were better able to induce Tr1 generation, while CD73^neg^ tumors were less effective in promoting Tr1 expansion. Thus, ADO emerges as an important target to consider in strategies designed to block tumor escape from the host immune system. 

## Figures and Tables

**Table 1:** Levels of cytokines or ADO measured in supernatants of Tr1TU73^+^ and Tr1TU73^neg^ or in supernatants of co-cultures of these Tr1 with autologous responder cells (RC) ^a^.

**A**							
**Cytokine**		**Tr1TU73^+^**		**Tr1TU73^+^** **+** **α,β-methylene-** **ADP**		**Tr1TU73^neg^**	
INFγ		4385 ± 1356		10,208 ± 3583		6918 ± 3060 *	
IL-10		632 ± 64		361 ± 9 *		151 ± 128 *	
TGFβ_1_		1115 ± 183		54 ± 26 *		56 ± 32 *	
**B**							
	**RC Alone**		**RC+ Tr1TU73^+^**		**RC + Tr1TU73^+^ +** **α,β-methylene-ADP**		**RC+ Tr1TU73^neg^**
IL-2	1063 ± 522		58 ± 8		409 ± 45 *		257 ± 121 *
INFγ	263 ± 63		8 ± 14		81 ± 49 *		135 ± 42 *
IL-10	BLD		282 ± 74		27 ± 8 *		70 ± 17 *
TGFβ_1_	BLD		1030 ± 171		249 ± 112 *		273 ± 111 *
Adenosine	4 ± 3		47 ± 11		20 ± 6 *		8 ± 2 *

^a^ Supernatants of Tr1 generated in co-cultures with TU CD73^+^ or TU CD73^neg^ cells were harvested after 24 h of stimulation with OKT3/CD28 mAb. Supernatants from different CFSE-based co-culture assays were harvested on day 5. Cytokine levels were determined by Luminex or ELISA. Results in pg/mL are means ± SD from 3 independent experiments for each co-culture. Asterisks * indicate significant differences at *p* ≤ 0.05 between Tr1TU73^+^ and Tr1TU73^neg^.
